# Skin Appendage Proteins of Tetrapods: Building Blocks of Claws, Feathers, Hair and Other Cornified Epithelial Structures

**DOI:** 10.3390/ani15030457

**Published:** 2025-02-06

**Authors:** Karin Brigit Holthaus, Julia Steinbinder, Attila Placido Sachslehner, Leopold Eckhart

**Affiliations:** Department of Dermatology, Medical University of Vienna, 1090 Vienna, Austria; karinbrigit@hotmail.com (K.B.H.);

**Keywords:** skin, keratinocytes, cornification, feather, claw, nail, scale, hair, reptiles, birds

## Abstract

Reptiles, birds, mammals and amphibians, together forming the clade tetrapods, have a large diversity of cornified skin appendages, such as scales, feathers, hair and claws. The skin appendages consist of dead epithelial cells that are tightly packed with specific structural proteins. Here, we review the molecular diversity and expression patterns of major types of skin appendage proteins, namely keratin intermediate filament proteins, keratin-associated proteins (KRTAPs) and proteins encoded by genes of the epidermal differentiation complex (EDC), including corneous beta-proteins, also known as beta-keratins. We summarize the current knowledge about the components of skin appendages with a focus on keratins and EDC proteins that have recently been identified in reptiles and birds. We discuss gaps of knowledge and suggest directions of future research.

## Simple Summary

Skin appendages, such as hair and feathers, are characteristic traits of mammals and birds, respectively. They are prototypes for a much broader range of skin appendages including also scales, claws, scutes and beaks, which develop in land-dwelling vertebrates. All these skin appendages have in common that they consist of dead cells which are tightly packed with specific proteins. This review provides an overview of the protein components of skin appendages characterized so far and identifies gaps of knowledge that need to be addressed by future studies. In particular, keratins and proteins encoded by genes of the so-called epidermal differentiation complex are highlighted as critical contributors to the architecture of feathers, scales and claws. As many skin appendage proteins have been predicted but not fully characterized yet with regard to their three-dimensional structure, interactions with other proteins and distribution in skin cells, they are important topics of ongoing research.

## 1. Introduction

**Figure 1:**
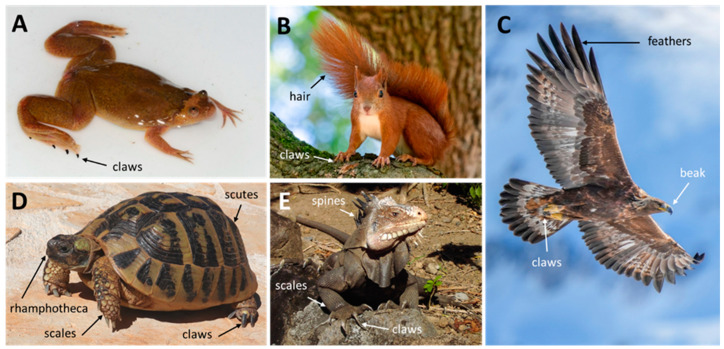
Cornified skin appendages of tetrapods. (**A**) Claws of an upland clawed frog (*Xenopus parafraseri*, Amphibia). (**B**) Hair and claws of a red squirrel (*Sciurus vulgaris*, Mammalia). (**C**) Feathers, claws and beak of a golden eagle (*Aquila chrysaetos*, Aves). (**D**) Rhamphotheca, scales, claws and scutes of a Hermann’s tortoise (*Testudo hermanni boettgeri*, Testudines). (**E**) Scales, spines and claws of an iguana (*Iguana delicatissima*, Lepidosauria). All images have previously been made publicly available under the CC BY-SA 4.0 license. Labels of skin appendages were inserted. Credits: Marius Burger (**A**), Rhododendrites (**B**), Giles Laurent (**C**), Palauenc05 (**D**), Hans Hillewaert (**E**).

The adaptation of vertebrates to different environments and lifestyles is associated with a large diversity of the skin and its appendages [1,2]. Hair and feathers are characteristic cornified skin appendages of mammals and birds, respectively. Scales cover the body of reptiles (sauropsids excluding birds), the legs of birds, the body of pangolins and the tails of some phylogenetically distinct mammals [3,4]. Claws are formed in all main groups of tetrapods, including some species of amphibians. Furthermore, specialized skin appendages, such as beaks, spines, scutes and horns, are present in subgroups of tetrapods. These skin structures consist of proteins, which vary in composition and arrangement among skin appendages and thereby determine their physical properties. Of note, skin glands, such as sweat glands and mammary glands, can also be considered skin appendages, although they are actually invaginations of the surface layer of the skin [5,6,7]. These glandular appendages are not a topic of this article. The aim of the present review is to provide an overview of protein components of cornified skin appendages, such as claws, feathers, hair and scales (Figure 1), which are located on the skin surface, and mediate direct interactions of tetrapods with their environment.

**Figure 2:**
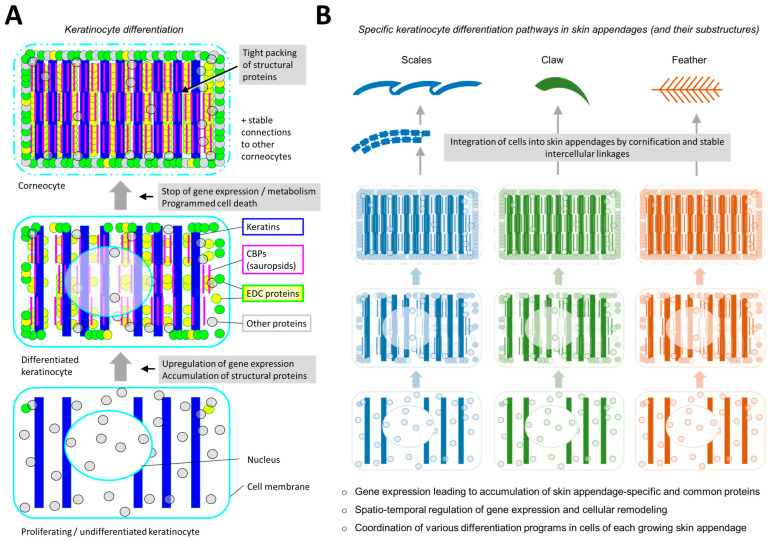
Structural proteins are the molecular building blocks of cornified skin appendages. (**A**) Schematic depiction of a generic keratinocyte differentiation program of sauropsids. The interior of an epithelial cell is progressively filled with proteins, including keratins, sauropsid-specific corneous beta-proteins (CBPs), epidermal differentiation complex (EDC) proteins and others, that are produced during terminal cell differentiation. Keratin-associated proteins (KRTAPs), which are expressed in skin appendage keratinocytes of mammals, are not included in this schematic. Note that the symbols do not represent the shape of the proteins. (**B**) Specific keratinocyte differentiation pathways are active in growing skin appendages. In this simplified schematic, the different colors indicate the expression of different sets of proteins in the different types of skin appendage. However, it is important to note that some proteins are expressed in more than one type of skin appendage. The proteins and their assembly into supramolecular structures determine the physico-chemical characteristics of each skin appendage.

The skin surface of tetrapods is formed by epithelial cells (keratinocytes) that proliferate in the basal layer of the epidermis, differentiate in the suprabasal layers and ultimately undergo a mode of programmed cell death in the course of forming cornified cell remnants known as corneocytes [8,9,10]. During differentiation, large amounts of cytoskeletal and other structural proteins are produced until they occupy most of the intracellular space. Cornification means the cross-linking of many proteins, the breakdown of organelles and the maintenance or stabilization of intercellular connections through desmosomes. The basic program of keratinocyte differentiation is modified in various ways, including the expression of different sets of structural proteins, in the epidermis, where newly formed corneocytes integrate into the cornified layer (stratum corneum), and in skin appendages, where corneocytes integrate into the cornified mature parts of the appendage (Figure 2) [11,12,13,14].

The development and growth of cornified skin appendages has been investigated mainly in mammals and sauropsids (reptiles and birds), whereas the development of skin appendages in amphibians has remained less well studied [1,3,15,16,17,18,19,20]. Many insights have been obtained into the signaling that leads to the pattern of skin appendage arrangement, the maintenance of stem cells, the interactions between epithelial and mesenchymal cells, the regulation of cyclic growth of some skin appendages such as hair and the sculpting of the shape of skin appendages [21,22,23,24,25]. All these processes are important for the growth and morphogenesis of skin appendages; yet, the function of the mature skin appendages depends primarily on their material components, which are mostly proteins (Figure 2). The protein composition of cornified skin appendages and the processes of producing and assembling different types of structural proteins are critical for the physical properties of skin appendages, and, accordingly, they are tightly controlled.

Before reviewing skin appendage proteins in detail, we have to mention that besides proteins, also other classes of molecules, and in particular lipids, contribute to the structure of cornified skin appendages. Lipids are estimated to constitute around 4% of the mass of hair fibers [26]. The protective function of the outermost layer, the epicuticle, of hair fibers depends on lipids. Likewise, lipids are constituents of feathers [27]. In contrast to the roles of lipids in the *stratum corneum* of the epidermis [28,29,30], the functions of lipids in cornified skin appendages have not yet been addressed in many research studies.

## 2. The Main Clades of Tetrapods Have Different Sets of Cornified Skin Appendages

### 2.1. Skin Appendages of Amphibians

Hard skin appendages are rare in amphibians, which comprise the subclades of anurans (frogs and toads), salamanders and caecilians. The epidermis of amphibians is soft and mucus-rich. It contains a stratum corneum consisting of a single layer of corneocytes [14,31]. Cornified claws develop in clawed frogs of the genera *Xenopus* and *Hymenochirus* and in clawed salamanders of the genus *Onychodactylus* [32,33]. Further sites of hard cornification are mouth parts (beaks) of tadpoles of many anurans [34,35,36], keratinized protrusions on the feet of spadefoot toads, nuptial pads on the limbs of several anurans and some salamanders such as the red-spotted newt [37,38] and nuptial spines of anurans [39].

### 2.2. Skin Appendages of Reptiles

Reptiles are a paraphyletic group of tetrapods comprising lepidosaurs (tuatara, geckos, lizards, snakes), turtles and crocodilians. Together with birds, which are phylogenetically the sister group of crocodilians, reptiles form the monophyletic clade of sauropsids. The skin has undergone significant diversification in reptiles, but cornified scales are characteristically the dominant skin appendages.

Scales are present in lepidosaurs, turtles and crocodilians. In lepidosaurs the whole body is covered by scales which are generally smaller, thinner and often more overlapping than the scales of crocodiles and turtles [40,41]. Typically, the outer scale surface is characterized histologically by the predominance of hard “beta layers” while the inner scale surface in contact with the next scale contains softer “alpha layers” [14,41,42,43,44]. Besides overlapping scales, tuberculated, rectangular, cycloid or keeled scales develop. Modifications of conventional scales manifest as cornified spines, frills, crests, tubercles and horns. The surface of squamate scales can display micro-ornamentations like spinules and setae, which are important for the interaction with the environment, for example, by improving the adherence to the substrate [45,46,47,48].

The particularity of crocodilian scales is that they are very thick and often reinforced by underlying matching osteoderms (bony dermal plates) [49,50]. In most species, this armor of protruding scales or scutes covers the dorsal part of the body and the keeled tail, whereas the ventral parts of the body and head have generally flat scales. Soft cornification is found in the narrow interscale or hinge regions of scales, while the larger thick outer surface of scales undergoes hard cornification [40,42]. Scales are also very prominent in turtles. Especially, the limbs of terrestrial turtles (tortoises) are covered by hard scales. Many aquatic turtles and soft-shelled turtles have relatively soft skin on the limbs, tail and neck [42,51].

Turtles (Testudines) are characterized by the development of a shell composed of the ventral plastron and the dorsal carapace. Both consist of a bony dermal part and an epidermal component. The latter is represented by scutes, which are considered large, thick and often flat scales [40]. Scutes are absent in soft-shelled and leatherback turtles [52].

Besides scales, claws are the second main type of cornified skin appendages of reptiles. They are conserved in turtles, crocodilians and most lepidosaurs. However, they are absent from snakes and other limbless squamates [53]. Furthermore, the tips of some toes of crocodilians are covered by scales instead of claws.

Turtles do not have teeth but a cornified structure comparable to the beak of birds. The corneous sheath of the upper and lower jaw is called the rhamphotheca [54].

### 2.3. Skin Appendages of Birds

Feathers and beaks are highly specialized hard epidermal appendages of birds. The feathers are structurally the most complex skin appendages of vertebrates [1,18,55,56,57]. They consist of a central shaft divided in a proximal calamus and distal rachis from which ramifications depart that are called barbs. The latter in turn have a shaft (ramus) and secondary ramifications named barbules [58,59]. Variations in form, size and rigidity of the aforementioned structural elements create six main feather types, namely semi- and filoplumes, bristles, down-, contour- and flight feathers. The functions of feathers are the support of flight, thermal insulation, visual communication, camouflage, tactile sensation and physical protection from the environment [60]. Like mammalian hair, feathers are cyclically regenerated from a follicle, which represents an extension of the skin epithelium with additional dermal elements [58].

The metatarsal region of bird feet is covered by overlapping scutate scales with alternating hard and soft cornification [61,62,63]. Of note, scutate scales are evolutionarily derived from feathers and only distantly related to reptilian scales [63]. Reticulate scales are found on the ventral part of the feet [62,64].

Cornified claws are consistently present on the hindlimbs of birds. Notably, claws develop also on the wings of turacos (*Musophagidae*) and the hoatzin (*Opisthocomus hoazin*) [65].

### 2.4. Skin Appendages of Mammals

Hairs are the characteristic skin appendages of mammals. The hair shaft is divided from the inside to the outside into medulla, cortex and cuticle, each formed by a distinct path of keratinocyte differentiation [66,67,68]. During growth of the hair follicle, the hair shaft is ensheathed by the inner and outer root sheath, which are also sites of unique paths of keratinocyte differentiation [66,69,70]. The main function of hair is thermal insulation, but it also functions in camouflage, communication and sensory perception [71,72,73,74]. Like feathers, hair is cyclically lost and regenerated [75], whereas other appendages continuously grow and are worn off over time.

Further cornified skin appendages of mammals are claws and their equivalents (nails and hooves), spines, scales and horns, which are characteristic for distinct subgroups of mammals. Spines of hedgehogs and echidnas and quills of porcupines have defensive functions. Their relation to hair is not fully understood [76]. A transcriptomic analysis of developing hair and spines showed differences in gene expression that need further investigations [77]. Scales of different morphologies cover large body parts of armadillos [78] and pangolins [79] and the tails of rodents [80], opossums and desmans [4]. The horns of ungulates are cornified epithelial sheaths over a bony outgrowth on the head, whereas horns of rhinos are purely of epithelial origin [81]. Whales of the genus *Mysticeti* develop a unique cornified skin appendage, namely baleen [82]. Notably, whales and dolphins (cetaceans) lack claws and hair with the exception of rudimentary vibrissae on facial skin [83].

## 3. Types of Proteins That Build Skin Appendages of Tetrapods

### 3.1. General Classification of Skin Appendage Proteins

Cornified skin appendages consist of remnants of cells that are packed with proteins to establish mechanically resilient structures. These so-called structural proteins can be subdivided in several types, of which the keratins, keratin-associated proteins and epidermal differentiation complex (EDC) proteins will be described in detail below. Another type of protein critical for the mechanical stability of skin appendages are the components of intercellular junctions (desmosomes), such as desmoplakin, desmogleins, desmocollins, plakoglobin and plakophilins [84,85,86,87,88]. A small portion of the total proteome of cornified skin appendages is made up of proteins without specific functions in its architecture. These non-structural proteins are involved in the synthesis of structural proteins and in the cellular metabolism up to the point when the cell undergoes cornification. Many of the non-structural proteins are degraded by autophagy in keratinocytes differentiating into corneocytes of hair shafts and nails [89,90,91] and probably also in other skin appendages. Proteins escaping intracellular degradation are integrated into corneocytes and contribute to the matrix between the cytoskeletal proteins of mature skin appendages.

The main types of structural proteins of hard skin appendages, but also of the epidermis, are encoded by genes of the type I keratin gene cluster, the type II keratin gene cluster [92,93,94] and the EDC [95,96,97,98,99,100]. While these gene clusters exist in all tetrapods, there are also important differences with regard to the number and type of genes present in different clades of tetrapods. First, the EDC of amphibians comprises only a few genes of unknown functions [100]. Second, only sauropsids have genes encoding corneous beta-proteins (CBPs), also known as beta-keratins, which have evolved within the EDC [1,14,42,61,86,101,102,103,104,105,106]. Third, only mammals have genes encoding keratin-associated proteins (KRTAPs or KAPs) located within the type I keratin gene cluster and at other chromosomal loci [68,107,108,109,110,111,112].

### 3.2. Keratins

Type I (acidic) and II (basic or neutral) keratins form hetero-dimers, which constitute the intermediate filaments of the cytoskeleton in keratinocytes of the epidermis and its appendages [113,114,115,116,117]. Both the type I and II keratins include proteins with a high cysteine content, and these keratins form monophyletic groups in molecular phylogenetics. As these cysteine-rich keratins are components of hair, they have been named “hair keratins” [67,116,118,119,120,121,122,123]. Hair keratins are not exclusively found in hair but also in nails [124,125], hooves, the horn of the rhinoceros [126], baleen of whales [127,128], scales and filiform papillae of the tongue [129,130,131,132]. Other keratins with lower numbers of cysteine residues are minor components of hard skin appendages in mammals.

Keratins play also central roles in the skin and skin appendages of sauropsids, where their expression was characterized for example in feathers and claws [14,133,134,135,136]. Many but not all clades of sauropsids have homologs of hair keratins [137,138,139,140]. Two additional cysteine-rich keratin types, named hard acidic sauropsid-specific (HAS) and basic sauropsid-specific (HBS) keratins, encoded by genes within the type I and II keratin cluster, respectively, exist in reptiles and birds [94,137]. Members of the HAS and HBS keratin families are conserved in all main taxa of sauropsids, and they contribute to hard skin appendage formation [94,137,141].

Keratins contain a central rod domain, which facilitates heterodimerization of one type I and one type II keratin by coiled-coil formation, and structurally flexible head and tail domains [142,143,144,145]. The keratin dimers are further assembled, presumably after anti-parallel arrangement of dimers in tetramers, into protofilaments and intermediate filaments [144,146,147,148,149]. Finally, the keratin intermediate filaments aggregate into dense bundles or tonofilaments, which constitute the cytoskeleton of keratinocytes [150]. Intermolecular disulfide bonds form at cysteine residues of the head, rod and tail domains [151]. The presence of unusually long tail domains containing multiple sequence repeats in specific keratins of sauropsids has led to the hypothesis that these domains mediate cross-linkage of intermediate filaments [152].

### 3.3. Keratin-Associated Proteins

The term keratin-associated proteins (KRTAPs, abbreviated KAPs in older publications) is used here to refer to proteins that are encoded by *KRTAP* genes of mammals [110] but not for other proteins that bind to keratins in mammals and other taxa. As *KRTAP* genes consist of only one exon and many of them are located in a tandem array within the type I keratin gene cluster of mammals, it was proposed that *KRTAP*s have evolved from a segment of an ancestral keratin gene [131]. Interestingly, some *KRTAP* genes, like hair keratin genes, are regulated by the transcription factor Hoxc13 [153].

KRTAPs do not have a conserved structural motif or a specific protein fold. Rather, they have amino acid sequences enriched in one or two amino acid residues, such as cysteine or tyrosine. KRTAPs are assumed to form the matrix between keratin intermediate filaments in hair and nails/claws of mammals.

### 3.4. Corneous Beta-Proteins

CBPs are the first sauropsid-specific epidermal proteins that have been discovered. For detailed information, we refer to several published reviews [105,154,155,156]. These proteins were initially called beta-keratins because they yield an X-ray pattern different from that of keratin intermediate filament proteins, then called alpha-keratins [42,157,158]. However, CBPs are phylogenetically not related to keratin intermediate filament proteins. CBP genes are located in the EDC of all sauropsids, with additional CBP genes being present on further loci in birds [96,98,131,159] and turtles [160,161].

A conserved segment of 34 amino acids gives rise to the pleated antiparallel beta-sheet structure characteristic for CBPs [61,105,155,158,159]. The amino acid sequences of the regions flanking the central beta-sheet are variable among different groups of CBPs [155,162]. The amino-terminal region is not only generally longer in lepidosaurs but also divided in two subdomains of sequences significantly different from the amino-terminal region of archosaur CBPs [155,162]. The carboxy-terminal regions of CBPs in lepidosaurs and archosaurs are similar in length and contain tyrosine and glycine-rich and arginine and cysteine-rich segments [155,162]. The so-called feather beta-keratins, now called feather CBPs, lack glycine and tyrosine-rich segments in the carboxy-terminal region [155,162]. The abundance of specific residues and the number and type of repeats vary among taxa, species and appendages and have been put in relation with the specific structural characteristics of the appendage [105,155]. Based on their predominant sites of expression, chicken CBPs have been classified as claw, scale, feather and keratinocyte CBPs [163,164,165,166].

Sauropsid CBPs interact through their characteristic beta-sheets through Van der Waals and hydrogen bonds [167,168,169]. CBP dimers form the building units of helical beta-filaments [155,167,170,171]. Lepidosaurs have a unique CBP, named Li-Ac40 in the green anole lizard and CBP1 in snakes, which contains four beta-sheets [172,173]. Modeling has suggested that the first two beta-sheets form a beta-sandwich in one filament and the second two beta-sheets form a beta-sandwich in another filament, thereby linking these filaments [171,174].

### 3.5. Epidermal Differentiation Complex Proteins Other than Corneous Beta-Proteins

EDC proteins are encoded by genes of the EDC and probably have a common evolutionary origin [98]. There are two types of EDC genes: single coding exon EDC genes (SEDCs) and genes with two coding exons. The latter encode either S100 proteins, which are not strictly skin-specific, or S100 fused-type proteins (SFTPs) [98,175,176]. Loricrin is a prototypical SEDC protein. It contains 40–55% glycine residues, which support the formation of flexible loops [177,178,179], and lysine and glutamine residues at its amino- and carboxy-terminus, which serve as sites of transglutaminase-mediated cross-linking to other proteins [98,177,180,181]. The best-characterized SFTPs are filaggrin, an important regulator of keratin aggregation and establishment of the barrier function in mammalian interfollicular epidermis [182,183], and trichohyalin, a constituent of scaffolding epithelia such as the inner root sheath of hair follicles [70,184].

Orthologs of loricrin, small proline-rich proteins and SFTPs exist in both mammals and sauropsids, but most EDC proteins are found either in mammals or in sauropsids, with further differences in the set of EDC proteins in different groups of sauropsids [96,98,161,173,185,186,187,188]. EDC proteins appear to play minor roles in mammalian skin appendages, whereas various EDC proteins are prominent components of skin appendages in sauropsids. The expression patterns of several EDC proteins have been partly characterized and will be discussed below. However, biomolecular and genetic data are not yet available for many EDC proteins of sauropsids.

## 4. Claw Proteins

### 4.1. Keratins Homologous to Hair Keratins in the Claws of Amphibians, Reptiles and Mammals

The human nail plate contains hair keratins such as keratin 31 (KRT31), KRT34, KRT81, KRT85 [124]. The corresponding genes are expressed in the nail matrix, which is the site of nail plate differentiation. Other keratins, such as KRT5 and KRT14, are expressed in the nail matrix as well as in regions of “soft” keratinization of the nail unit, such as the proximal nail fold (eponychium) and under the tip of the nail (hyponychium) [124,189]. KRT75 and KRT6 with their dimerization partners KRT16 and KRT17 [189,190,191] are expressed in the nail bed, which does not contribute to the mature nail plate.

**Figure 3:**
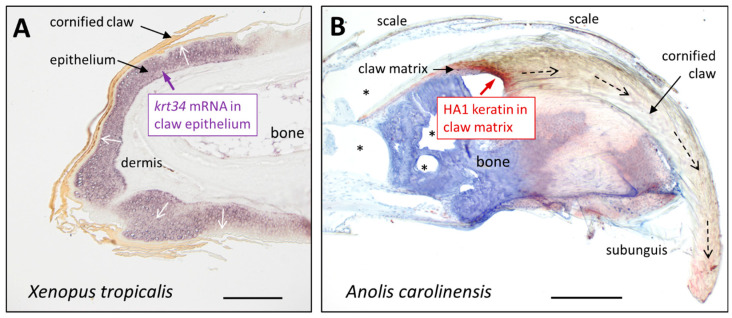
Homologs of hair keratins are expressed in the claws of amphibians and reptiles. (**A**) mRNA in situ hybridization (violet) of *krt34* in a hindlimb toe of *X. tropicalis* [193]. (**B**) Immunohistochemical staining (red) of hard acidic keratin 1 (HA1) and counterstaining with hematoxylin (blue) in a toe of *A. carolinensis* [138]. Sectioning artifacts are marked by asterisks. The white arrows indicate the direction of epithelial cell differentiation in (**A**). Arrows with discontinuous lines indicate the direction of claw growth in (**B**). Scale bars: 200 µm (**A**) and 500 µm (**B**). The image in (**A**) is reproduced from Figure 1B of a paper recently published under a Creative Commons CC-BY 4.0 license (open access) [193]. The image in (**B**), originally published in Figure 1A of Alibardi L, Jaeger K, Dalla Valle L, Eckhart L. Ultrastructural localization of hair keratin homologs in the claw of the lizard *Anolis carolinensis. J Morphol*., published by John Wiley and Sons, Inc. 2011 [138], is reproduced with permission from John Wiley and Sons.

Homologs of hair keratins have been found in the claws of reptiles and amphibians, revealing their evolutionarily ancient function in skin appendage formation [137,192,193]. In the claws of the hindlimbs of the African clawed frog (*Xenopus tropicalis*), a homolog of hair keratin 34 was identified by in situ hybridization (Figure 3A), quantitative RT-PCR and mass spectrometry-based proteomics [193]. Further confirmation of this result was obtained on the gene level by claw-specific expression of *krt34*. Homologs of hair keratins were also detected in toes of the axolotl [193] and in the toe pads of the tree frog *Hyla cinerea* [192], suggesting that hair keratin homologs are not restricted to cornified claws in non-mammalian tetrapods but rather linked to the toes. The transcription factor Hoxc13, which is expressed in the posterior body (tail) and distal portions of the limbs, is required for the expression of hair keratin homologs in the claws of *X. tropicalis* and in the claws/nails and hair of mammals [193,194,195].

Reptilian homologs of hair keratins, named hard acidic keratin 1 (HA1) (Figure 3B) [137,138] and hard basic keratin 1 (HB1), have been shown by immunohistochemistry to be components of claws in the green anole lizard [137]. In addition, the mRNA of these genes was detected in clawed toes. Immunogold electron microscopy revealed HA1 and HB1 in the corneous and precorneous layers of the claw [138]. Alternative names of HA1 and HB1 are KRT36L1 and KRT84L4, respectively [94].

Loss of claws and hair [4,73,196] has led to loss of most hair keratins in whales and dolphins [197]. Likewise, in reptiles, the absence of claws in snakes and worm lizards is associated with the lack of hair keratin homologs [94,139,140].

### 4.2. Keratins Not Orthologous to Hair Keratins

Besides hair keratin homologs, various amounts of other keratins are present in the claws of different clades of tetrapods. In mouse nails and *Xenopus* claws, keratins not belonging to the hair keratin clades are much less abundant than hair keratins [89,198]. KRT42 and KRT124, which lack orthologs in humans, are the most abundant keratins in the hoof lamellar tissue of horses [199]. Proteomic analysis revealed several keratins in the claws of the chicken [86], which lack genes for type I keratins [94]. Because the protein sequence database used for this study did not include all keratins and in particular the cysteine-rich HAS and HBS keratins [94], the keratin composition of avian claws requires further studies. Similarly, it will be interesting to investigate the claws of turtles, which also lack homologs of type I hair keratins [94].

### 4.3. Corneous Beta-Proteins of Claws

Claws of sauropsids contain CBPs. A group of CBPs was reported to be mainly expressed in chicken claws, leading to the name claw beta-keratins (now called claw CBPs) or “claw keratins”, although these proteins are not true keratins [163,164,165]. Expression of CBPs was also detected at the mRNA level in the claws of chickens [103] and alligators [104]. CBPs are expressed both in the claw (unguis) and subunguis of the chicken [136]. The proteomic analysis of chicken claws confirmed the presence of CBPs [86]. In the green anole lizard, two glycine and cysteine-rich CBPs were reported to be specifically expressed in claws [172].

### 4.4. Epidermal Differentiation Complex Proteins of Claws

A proteomic analysis of chicken claws found several EDC proteins, of which Epidermal Differentiation protein containing Y (tyrosine) Motif 1 (EDYM1) and Epidermal Differentiation protein containing a WYDP (tryptophane, tyrosine, aspartic acid, proline) Motif (EDWM) were highly abundant [86,98]. The genes for EDYM1 and Epidermal Differentiation proteins containing Cysteine Cysteine motifs (EDCCs) have been lost in snakes, suggesting that they have become dispensable after the evolutionary loss of limbs and claws [173].

Cornulin, trichohyalin and the sauropsidian trichohyalin-like protein scaffoldin are expressed in the nail isthmus and subunguis but not in the cells contributing to the mature nail plate or claw [200].

## 5. Scale Proteins

### 5.1. Keratins of Scales

Keratins were detected by proteomic analysis [86], immunolabeling [201,202] and mRNA in situ hybridization [64,136] in scales of different sauropsids. In geckos, two keratins were identified in the adhesive toe pads [102]. As HAS and HBS keratins were characterized only recently [94], their role in scale cornification was not considered in earlier studies.

**Figure 4:**
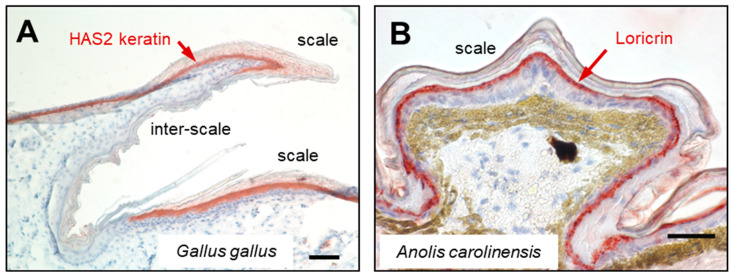
Keratins and loricrin contribute to the protein composition of scales in different sauropsids. (**A**) Immunohistochemical staining (red) of hard acidic sauropsid-specific 2 (HAS2) keratin in scutate scales on the leg of the chicken (*G. gallus*) [141]. (**B**) Immunohistochemical staining (red) of loricrin in scales of the green anole lizard *A. carolinensis* [98]. Scale bars: 50 µm (**A**) and 40 µm (**B**). The images are reproduced from papers published under the Creative Commons CC-BY 4.0 license (open access) [98,141].

Using a specific antibody, HAS2 keratin, also referred to as keratin 9-like cysteine-rich 2 (KRT9LC2), was detected by immunohistochemistry in the hard scutate scales but not in the soft interscale (hinge) regions on chicken legs (Figure 4A) [141]. This pattern of expression was confirmed by single cell transcriptomic analysis [141]. The HAS2 keratin is expressed in scutate scales but not in reticulate scales and soft skin between feather follicles.

### 5.2. Corneous Beta-Proteins of Scales

CBPs are components of the different types of scales in sauropsids. Mass spectrometry-based proteomics detected CBPs in scutate scales of the chicken [86]. An antibody against a feather CBP was used to localize CBPs in developing scutate scales of both chicken and crocodiles, and an antibody against claw CBPs was used to localize CBPs in crocodile scutes [203,204]. In another study, crocodilian scale CBPs were immuno-localized at both the ultrastructural and histological level [201]. Early work detected CBP expression in the subperiderm of scutate and reticulate scales of embryonic chickens and in scutate scales of adult chickens [205]. Differential regulation of five CBPs was demonstrated by in situ hybridization in embryonic reticulate and scutate scales of chicken on embryonic days E14 and E16 [136]. CBPs are expressed on the outer but not, or at much lower levels, on the inner surface of scutate scales.

In the scales of lizards and geckos, CBPs were detected by immunostaining, immunogold transmission electron microscopy and mRNA in situ hybridization [102,206,207,208,209]. Interestingly, CBPs were reported to be components of toe pad lamellae, considered modified scales with setae, which are utilized by geckos for climbing vertical surfaces [209].

### 5.3. Epidermal Differentiation Complex Proteins of Scales

The glycine-rich EDC protein loricrin has been characterized initially in mammals, where it is the main component of the cornified envelope in the stratum corneum [210], but homologs of loricrin are also present in sauropsids [98,187]. Loricrin was found by proteomic analysis in chicken scutate scales [86,98]. In scales of the green anole lizard, loricrin 1 was detected by immunohistochemistry (Figure 4B). By immunogold transmission electron microscopy, loricrin 1 was localized specifically to the lacunar cells of the alpha-layer [211]. Proteomic analysis showed that an isoform of loricrin, EDWM, Epidermal Differentiation protein containing Cysteine Histidine motifs 4 (EDCH4) and Epidermal Differentiation protein containing glutamine (Q) repeats (EDQrep) are abundant in chicken scutate scales [98].

## 6. Feather Proteins

### 6.1. Keratins (Keratin Intermediate Filament Proteins)

A proteomic analysis of feathers revealed the presence of keratins besides CBPs and other proteins [86,98]. Most of the keratins predicted in chicken were found to be expressed in the regenerating feather epithelia comprising rachis, feather branches and sheath [135]. Moreover, in situ hybridizations were used to localize the expression of keratins in feathers during development [135,136]. The ultrastructural localization of keratins was determined by immunogold electron microscopy [55,212].

**Figure 5:**
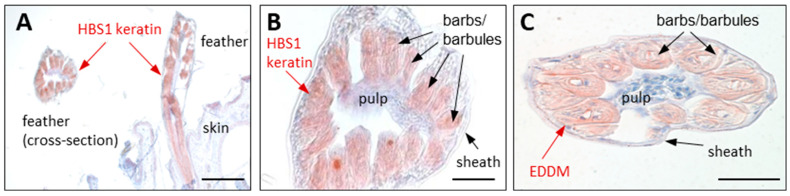
Keratins and the Epidermal Differentiation protein containing DPCC Motifs (EDDM) contribute to the protein composition of feathers. (**A**,**B**) Immunohistochemical staining (red) of HBS1 keratin, also known as KRT78LC1, in feathers of a chicken (*G. gallus*) during embryonic development [94]. (**C**) Immunohistochemical staining (red) of EDDM in feathers of a chicken during embryonic development [213]. Scale bars: 200 µm (**A**), 50 µm (**B**) and 50 µm (**C**). The images are reproduced from papers published under the Creative Commons CC-BY 4.0 license (open access) [94,213].

Using an antibody raised against a specific epitope of chicken HBS1 keratin, also referred to as keratin 78-like cysteine-rich 1 (KRT78LC1), this keratin was detected in the barbs and barbules of developing chicken feathers (Figure 5A,B) [94]. HBS1 showed feather-specific gene expression at the mRNA level [94]. *KRT75* mRNA was detected in the rachis and the ramus of chicken feathers, and a mutation of the corresponding gene was shown to cause a defective structure of the rachis, underlying the “frizzle” feather trait [134].

### 6.2. Feather Corneous Beta-Proteins

Feathers contain a large variety of CBPs, indicating that they are major protein components of these skin appendages [103,133,135]. Seven CBPs were localized by in situ hybridization in regenerating feathers, revealing differential expression in two feather types and during development [135]. Both CBPs encoded by genes in the EDC on chicken chromosome 25 and CBPs encoded by genes on other loci are expressed in feathers [25]. The importance of the structural role of CBPs was confirmed by suppressing the expression of CBPs by antisense RNA, leading to disturbed feather morphogenesis [136]. CBPs were also localized at the ultrastructural level in feather cells [55,214]. Proteomic analysis showed higher numbers of CBP-derived peptides in feathers than in claws, beak and scales [86].

### 6.3. Epidermal Differentiation Complex Proteins of Feathers

The expression of EDC genes, besides genes encoding CBPs, in growing feathers has been recognized only in the past fifteen years. Although the analysis of EDC gene expression in feather follicles has remained incomplete, several EDC proteins are now established as components of mature feathers.

The Epidermal Differentiation protein starting with an MTF (methionine, threonine, phenylalanine) motif and rich in Histidine (EDMTFH) belongs to the family of Epidermal Differentiation proteins rich in Aromatic Amino acid residues (EDAAs), which comprises also orthologs in crocodiles and turtles [161,185]. This bird protein was originally reported under the name histidine-rich protein or fast protein [215]. Later, the amino acid sequence prediction was corrected and the protein was renamed to consider the localization of the corresponding gene in the EDC [98]. EDMTFH was detected by immunohistochemistry in the barbs and barbules of growing chicken feathers [216,217].

The Epidermal Differentiation protein containing DPCC (aspartic acid, proline, cysteine, cysteine) Motifs (EDDM) and the Epidermal Differentiation Cysteine-Rich Protein (EDCRP) are highly cysteine-rich proteins with 152 (23%) and 140 (36%) cysteine residues, respectively, in the chicken [213,218]. The sequence similarity of EDCRP with the mammalian cysteine-rich or high/ultrahigh sulfur KRTAPs suggests convergent evolution of cysteine-rich proteins in feathers and hair [108,218,219]. Notably, the cysteine residues are the sites of disulfide cross-linking between hair KRTAPs and keratins in human hair and nail [112,151,220]. *EDCRP* gene expression was localized by mRNA in situ hybridization to the barbule cells of feathers in embryonic chickens (E18) [218]. An ortholog of EDCRP has been identified in crocodilians, the phylogenetic sister group of birds, and a protein with sequence similarity exists in squamates [98,185,186,187], suggesting that the sequence features of EDCRP are not feather-specific.

EDDM was localized by immunolabeling in the feather barbs and barbules of embryonic chicken (Figure 5C). Quantitative RT-PCR confirmed high expression of the *EDDM* gene in the developing feathers of embryonic chickens [213]. *EDDM*-like genes were also identified in crocodilians [185,213]. Mass spectrometry-based proteomic analysis confirmed the presence of EDDM in chicken feathers [86,98].

## 7. Turtle Scute Proteins

### 7.1. Keratins of the Turtle Shell

Keratins are expressed in the living epidermal layers and the hinge regions of scutes [214,221,222]. An antibody raised against a type I keratin of the spiny softshell turtle (*Apalone spinifera*) was used for comparative immunolabeling studies of soft-shelled and hard-shelled turtles [214]. The methodology for the proteomic analysis of hard cornified skin appendages of birds [86] may be suitable for obtaining unbiased insights into the protein composition of scutes of turtles.

### 7.2. Corneous Beta-Proteins of Scutes

A series of experimental studies have indicated that CBPs are the main components of the hard scutes of the turtle shell [154,223,224]. CBPs of the turtles were identified before whole genome sequences became available [225]. Most of the initially cloned CBPs were detected in the epithelium which forms the cornified part of the shell [225]. More evidence of the presence of CBPs in the shell came from a study with an antibody against a broad range of CBPs in a hard-shelled turtle [221]. Another study focused on the seasonal variation of CBPs in scutes, which is correlated to the growth and resting phases of the turtle skin [222]. With a generic CBP antibody, CBPs were also immunolocalized in the shell and skin of a soft-shelled turtle [51]. Two CBP genes, one located in the EDC and the other at a different locus, were reported to be expressed in the carapace of the turtle shell [161]. Some genes for CBPs of the turtle shell are located outside of the EDC, reminiscent of CBP genes implicated in feather morphogenesis [133,135,160,161].

### 7.3. Epidermal Differentiation Complex Proteins in Scutes of the Turtle Shell

Epidermal Differentiation proteins rich in Proline, Cysteine and Valine (EDPCVs) are encoded by EDC genes of turtles [161]. mRNAs for these proteins were detected predominantly in the carapace, while expression of another SEDC gene named *EDQM7* was detected in the plastron of the European pond turtle (*Emys orbicularis*) [161]. In agreement with the hypothesis that EDPCVs have functions in the scutes of the turtle shell, the Chinese soft-shelled turtle (*Pelodiscus sinensis*) has lost not only the hard scutes of the shell but also most *EDPCV* genes so that only 4 *EDPCV* genes exist in this species as compared to 15 *EDPCV*s in the painted turtle (*Chrysemys picta*) [161].

## 8. Proteins of Beaks

### 8.1. Beak Keratins

The proteome of the cornified portion of the chicken beak contains keratins as one of the predominant protein types besides desmosomal proteins [86]. The composition of the beak is more similar to that of claws than those of feathers and scales [86].

The interpretation of the proteomics results is complicated by the fact that some of the protein sequences were incomplete or not present in the database available at the time of this study. For example, one of the most abundant proteins in the beak (Uniprot entry E1BZZ9, corresponding to UniParc entry UPI0000ECA069) is a fragment of the HAS1 keratin, also named KRT9LC1 [94], which belongs to the sauropsid-specific cysteine-rich type I keratins. In another study, four keratins were localized by in situ hybridization in the embryonic chicken beak [136]. Like the beak of birds, the rhamphotheca of turtles contains keratins [54].

### 8.2. Corneous Beta-Proteins of Beaks

The beak of the main model species of birds, that is, the chicken, was reported to contain CBPs [86]. As mentioned before with regard to keratins, the annotation of CBPs was incomplete in these data. Consequently, cornified beaks of birds should be investigated in proteomic analyses using the currently available CBP sequence predictions. Three CBPs were detected by in situ hybridization in the beak of chickens [136]. Many years earlier, so-called scale CBPs were found to be expressed in the cornified part of the developing beak of a chicken [226]. CBPs are also major components of the rhamphotheca in turtles [54].

### 8.3. Epidermal Differentiation Complex Proteins of Beaks

EDWM, EDYM1, EDCH4 and EDMTF4 were identified by proteomics in the beak of chickens [98]. At the mRNA level, *EDYM1* was most abundant in the developing chicken beak as compared to other tissues [98]. The mRNAs of several other EDC genes were detected in the beak, but levels and localization of the corresponding EDC proteins have not been determined yet.

## 9. Hair Proteins

### 9.1. Hair Keratins

Keratins are the quantitatively dominant components of hair [67,90,118,227]. Specific hair keratins are expressed in the concentric layers of the hair shaft. The human hair cuticle contains KRT32, KRT35, KRT39, KRT40, KRT82 and KRT85, whereas the cortex contains KRT31, KRT33, KRT34, KRT35, KRT36, KRT39, KRT81, KRT83, KRT85 and KRT86 [129]. The innermost part of the human hair shaft, the medulla, contains a broad range of hair keratins and epithelial keratins [228]. Hair keratins are largely conserved in mammalian species, but there are also differences even between humans and their closest relatives among primates [229]. Besides human and mouse hair, sheep wool was studied intensively [220,230].

### 9.2. Keratin-Associated Proteins in Hair

KRTAPs are the second most abundant type of proteins in hair [231]. The high/ultrahigh cysteine and high glycine-tyrosine subfamilies of KRTAPs were identified in the human hair shaft [110,219,232]. These proteins are also present in other mammals but not in reptiles or birds [112,233]. At least 27 KRTAPs have been identified in sheep wool, and their expression pattern is similar in human hair [219,234].

### 9.3. Epidermal Differentiation Complex Proteins in Hair

Trichohyalin, encoded by the gene *TCHH*, is expressed in the differentiating medulla, located in the center of several but not all types of hair. Similarly, trichohyalin is involved in the differentiation of the inner root sheath of the hair follicle [184,235], which, however, is not a part of the mature hair shaft [236]. Involucrin was detected by immunolabeling in the medulla [237]. Other EDC proteins contribute probably little if anything to the proteome of hair shafts.

## 10. Cross-Linking of Proteins in Cornified Skin Appendages

### 10.1. Protein Cross-Linking by Disulfide Bond Formation Between Cysteine Residues

The hardness of cornified skin appendages derives largely from the massive cross-linking of structural proteins through covalent bonds. Disulfide bonds and isopeptide bonds are the two types of inter-protein connections in skin appendages. Disulfide bonds between cysteine residues are formed by a non-enzymatic reaction. An involvement of enzymatically catalyzed oxidation in disulfide bond formation during keratinocyte cornification has been proposed [238]. These cross-links are prevalent between keratins and between keratins and KRTAPs [151,239,240]. Disulfide bond formation is also the main type of protein cross-linking in hard skin appendages of sauropsids [94,241]. Presumably to support this cross-linking, several EDC proteins have an extremely high abundance of cysteine residues [187,213,218].

### 10.2. Protein Cross-Linking by Transglutamination

Transglutaminases (TGMs) are calcium-dependent enzymes that catalyze the formation of N^ε^-(-glutamyl)lysine isopeptide bonds between glutamine and lysine residues of proteins. TGM1, TGM3 and TGM5 are involved in human and murine epidermal cornification [9,242]. Transglutamination of multiple proteins generates the cornified envelope in differentiated keratinocytes that integrate into the stratum corneum [9,242,243]. TGM1 exerts most of the transglutaminase activity in mammalian keratinocytes, where it cross-links loricrin, small proline-rich proteins (SPRRs) and other proteins [181,183]. It has been proposed that TGM 1 and 5 initiate protein cross-linking and TGM3 contributes to the reinforcement of the developing envelope [9].

TGMs are partly conserved in vertebrates [198,244,245,246,247], but information about the roles of these enzymes outside the mammalian clade is scarce. By immunoblotting, TGM was identified in the skin of sauropsids [246]. Histochemical studies suggested that TGMs are almost absent in the epidermal layer with hard cornification (beta layer) such as scales but present in the layer with soft cornification (alpha layer) [241]. In the chicken, TGM activity was localized by in situ fluorescence labeling in the claw matrix and in feather follicles [247]. Gene expression data suggest predominant roles of TGM9 in the epithelial eggtooth of birds and in claws of lizards [198].

## 11. Conclusions and Perspectives

An increasing body of evidence indicates that the specific protein composition is critical for the function of cornified skin appendages. Although many proteins of skin appendages have been identified, the knowledge about the molecular architecture of skin appendages is incomplete. To address important knowledge gaps, we propose the following directions of future research: 1. We need a comprehensive catalog of protein components of all types of skin appendages. This is especially necessary for non-mammalian cornified skin structures. A wider range of species of both mammals and non-mammalian tetrapods should be investigated to test whether conclusions obtained in model species can be generalized. 2. Protein components of skin appendages should be localized in situ. Immunolabeling with validated antibodies, spatial proteomics and supportive investigations of the corresponding mRNAs by in situ hybridization or single cell transcriptomics will be very informative. 3. The interactions between proteins and particularly the covalent connections between proteins should be determined as precisely as possible. The currently available methods are technically challenging [151,183,240] and may need to be supported by new methods. 4. The spatio-temporal control of gene expression by transcription factors and other regulators is a topic of studies that will shed new light onto epithelial differentiation during skin appendage growth. 5. Targeted gene manipulation studies in vivo and modifications of gene expression in ex vivo organ cultures [248] are powerful methods for the functional analysis of individual skin appendage proteins. However, these methods are not well established for reptiles and birds, and the skin appendages of these taxa are the least characterized. Accordingly, many new insights can be expected if technological advances are achieved. 6. The three-dimensional structures of many skin appendage proteins need to be defined at high resolution. To determine the structure of CBPs should be one of the research priorities. 7. Further investigations should be carried out to determine how the structural proteins affect the physical properties of skin appendages. These studies will not only help to understand the natural functions of skin appendages but may also lead to the use of proteins or design principles of skin appendages in technical applications [249].

## Data Availability

Not applicable.
